# Subluxation-induced liner adhesion and the effect of impaction to prevent liner dislocation in ceramic hip arthroplasty

**DOI:** 10.1302/2046-3758.156.BJR-2025-0615.R1

**Published:** 2026-06-17

**Authors:** Maximilian Uhler, Mareike Schonhoff, Sebastian Jäger, Therese Bormann, J. Philippe Kretzer

**Affiliations:** 1 Research Center of Biomechanics and Implant Technology, Department of Orthopaedics, Heidelberg University Hospital, Heidelberg, Germany

**Keywords:** Hip arthroplasty, Ceramic-on-ceramic, Adhesion forces, subluxations, hip arthroplasty, total hip arthroplasty, strength, acetabular component, Pearson correlation coefficient, femoral head, hip joint, serum, hip

## Abstract

**Aims:**

Ceramic-on-ceramic bearings are commonly used in total hip arthroplasty. The long-term clinical results show low revision rates. However, there might be the possibility of liner dislocation during clinical use, due to adhesion forces occurring in the bearing.

**Methods:**

In this study, the adhesion forces during subluxation of the ceramic head from the liner were measured. Two different implant systems (Mathys SeleXys and DePuy Synthes Pinnacle) with two different head sizes (28 mm and 36 mm) were studied. Additionally, the liner-to-cup fixation forces with three different liner sizes (44 mm, 52 mm, and 64 mm) were analyzed to correlate the adhesion forces with the liner fixation force. Three different assembly forces (1 kN, 2 kN, and 4 kN) were considered.

**Results:**

There was a significant difference in the adhesion forces between Mathys SeleXys and Depuy Synthes Pinnacle bearings at a head size of 28 mm. However, no significant difference was found for the 36 mm head size. The maximum adhesion force reached 265 N. The liner fixation forces varied depending on the assembly force. For an assembly force of 1 kN, the fixation forces varied between 95 and 385 N, which is partly below the corresponding adhesion force. For assembly forces of 2 kN or more, the liner fixation force was always above the adhesion forces.

**Conclusion:**

A low liner impaction force can lead to liner dislocation due to possible adhesion forces between the ceramic head and the ceramic liner. Sufficient liner impaction forces of more than 2 kN should be applied to prevent the risk of adhesion-related liner extractions.

Cite this article: *Bone Joint Res* 2026;15(6):696–704.

## Article focus

- Investigation of adhesion forces between the ceramic head and liner in hip arthroplasty.
- Analysis of fixation strength of ceramic liners depending on assembly force.
- Evaluation of whether implant design parameters could influence the liner stability.

## Key messages

- Adhesion forces can be sufficient to cause liner dislocation, especially with low assembly forces.
- Assembly forces over 2 kN are recommended to reduce the risk of liner dislocation in the studied implants.
- The implant design significantly affects the fixation strength.

## Strengths and limitations

- Extensive biomechanical testing was conducted under standardized laboratory conditions.
- Comparative analysis of two implant systems.
- Bovine serum was used, which limits comparison with human synovia; a limited number of implant designs were used; and testing was carried out only in the optimal pulling direction.

## Introduction

The use of ceramic-on-ceramic bearings in total hip arthroplasty results in the lowest amount of wear compared to other material combinations.^1-3^ In spite of this, fewer than 10% of primary hip implantations are performed with this material combination in the UK, USA, and Germany.^4-6^ The reason for this may lie in clinical complications with these bearings.^7^ In a study by Brandt et al,^8^ approximately 17% of all revised ceramic-on-ceramic bearings (n = 35) underwent revision surgery due to squeaking noises or fractures of the ceramic components. Clinical failures are often associated with a surgical malalignment of the ceramic liner.^9-12^ In addition to the malalignment during impaction,^13-15^ factors such as acetabular component deformation^10,11^ or entrapment of soft-tissue are reported,^11^ which can also result in a malalignment or incomplete seating of the ceramic liner in the cup. This can lead to uncontrolled stress peaks, edge loading, or even breakage of the ceramic components.^9-12,15^ The ceramic liner breaks about 14 times more often than the ceramic head.^9^ If a ceramic component breaks, replacement is critical, as residual ceramic particles can cause excessive third-body wear.^16^ Only ceramic-on-polyethylene bearings result in low wear after revision.^17^

A factor that has not yet been considered as a cause for incorrect liner positioning is a possible extraction of the liner from the cup due to adhesion forces between the liner and head. In vitro studies have shown that relevant adhesion forces can occur between the bearing partners during dislocation of the joint.^18^ In addition to the head diameter,^9,19^ the speed of dislocation is also a decisive factor for liner dislocation.^18^ Apart from the design and external factors, the composition of the synovial fluid may also have an influence on the adhesive force.^19^

**Fig. 1:**
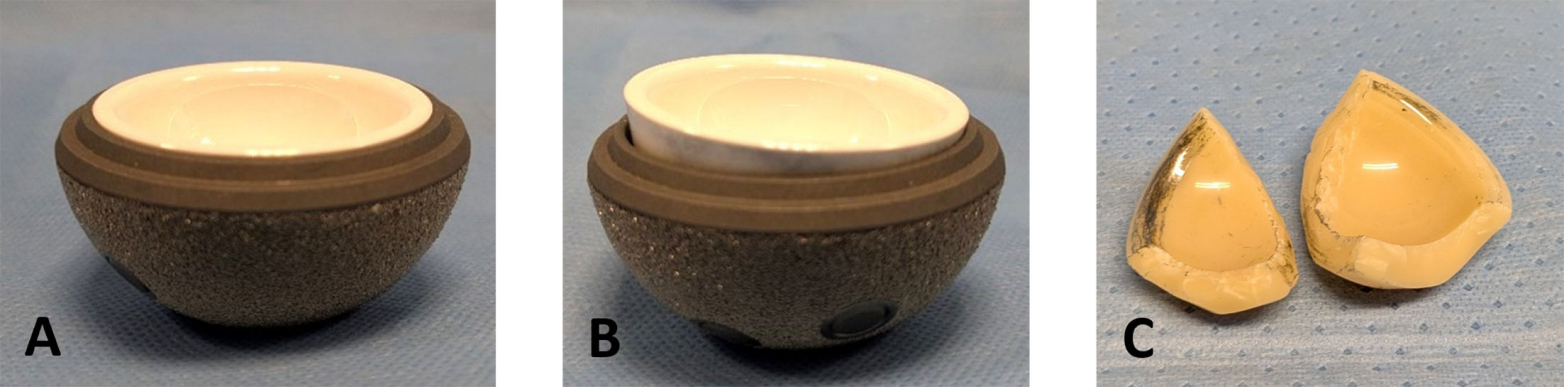
a) Well-aligned ceramic liner in cup. b) Dislocation of liner in cup. c*)* Fractured liner from a clinical retrieval*.*

Subluxations of the joint can occur as a result of various factors, such as different daily activities.^20-22^ They can also occur consciously or unconsciously during surgery when testing joint stability, or in the early postoperative phase when mobilizing the hip joint or during physiotherapy.^23,24^ If the joint is slightly subluxated, adhesive forces between the bearing partners may rise and provoke a liner extraction from the cup even if the components were surgically well aligned (Figure 1a). During the relocation of the liner, a malalignment in the cup can occur (Figure 1b). Due to the malalignment, the ceramic liner may fracture later or in the long term, if the joint is fully loaded (Figure 1c).

As the fixation strength of a liner in the cup depends on the assembly force, it remains unclear how strongly the liner needs to be impacted to prevent an adhesion-driven liner extraction. Furthermore, the impaction force varies depending on the operator.^25,26^ So far, liner dislocation associated with adhesion forces has been investigated on bearing partners with polyethylene liners and metal-on-metal bearings.

The aim of this study was to investigate the fixation strength of the ceramic liner and the possibility of loosening the liner due to adhesion forces acting in the bearing, which can occur during slight bearing dislocation. The following research questions are addressed within the study: 1) what adhesive forces occur in ceramic-on-ceramic bearings during simulated subluxation?; 2) are the adhesion forces influenced by the head diameter combined with the clearance between head and liner or the distraction speed?; 3) how are assembly force, cup size, and liner taper angle correlated to the fixation strength of ceramic liners?; and 4) is there an assembly force to be recommended to prevent liner dislocation?

## Methods

To investigate the effect of liner-head adhesion, two different implant systems were analyzed: the Mathys SeleXys PC Multi-Hole (Mathys, Swiss) and the DePuy Synthes Pinnacle Multi-Hole Porocoat Gription (DePuy Synthes, USA). Each system was tested in three distinct sizes, which are shown in Table I. The taper angle between the liner and the cup differs between the two manufacturers. For the Mathys implant, the angle was measured at 9.5°, whereas for the DePuy implant it was found to be 5.0°.

**Table I.** Implant sizes*.*

Implant system	Size	Cup size (Ø in mm)	Liner size (Ø in mm)	Head size (Ø in mm)
Mathys SeleXys	Small	44	28	28
	Medium	52	36	36
	Large	64	36	36
DePuy Synthes Pinnacle	Small	44	28	28
	Medium	52	36	36
	Large	64	36	36

Measurements of the liner, as well as the head size, were performed using a 3D coordinate measuring machine (CMM) (Mahr Multisensor MS 222; Mahr GmbH, Germany) with an accuracy of ± 2.3 μm. To enable an evaluation that combines the head size and the clearance, a ball-on-plane model was adopted and the equivalent radius (R') was determined. The clearance represents the actual radial difference between the head and the inlay. R' is used as a combined parameter to characterize the gap geometry between the head and the inlay. R’ was calculated as follows:

R′=R_1_*R_2_/R_2_−R_1_

where $R_{1}$ is the head radius and $R_{2}$ is the cup radius.^27^

**Fig. 2:**
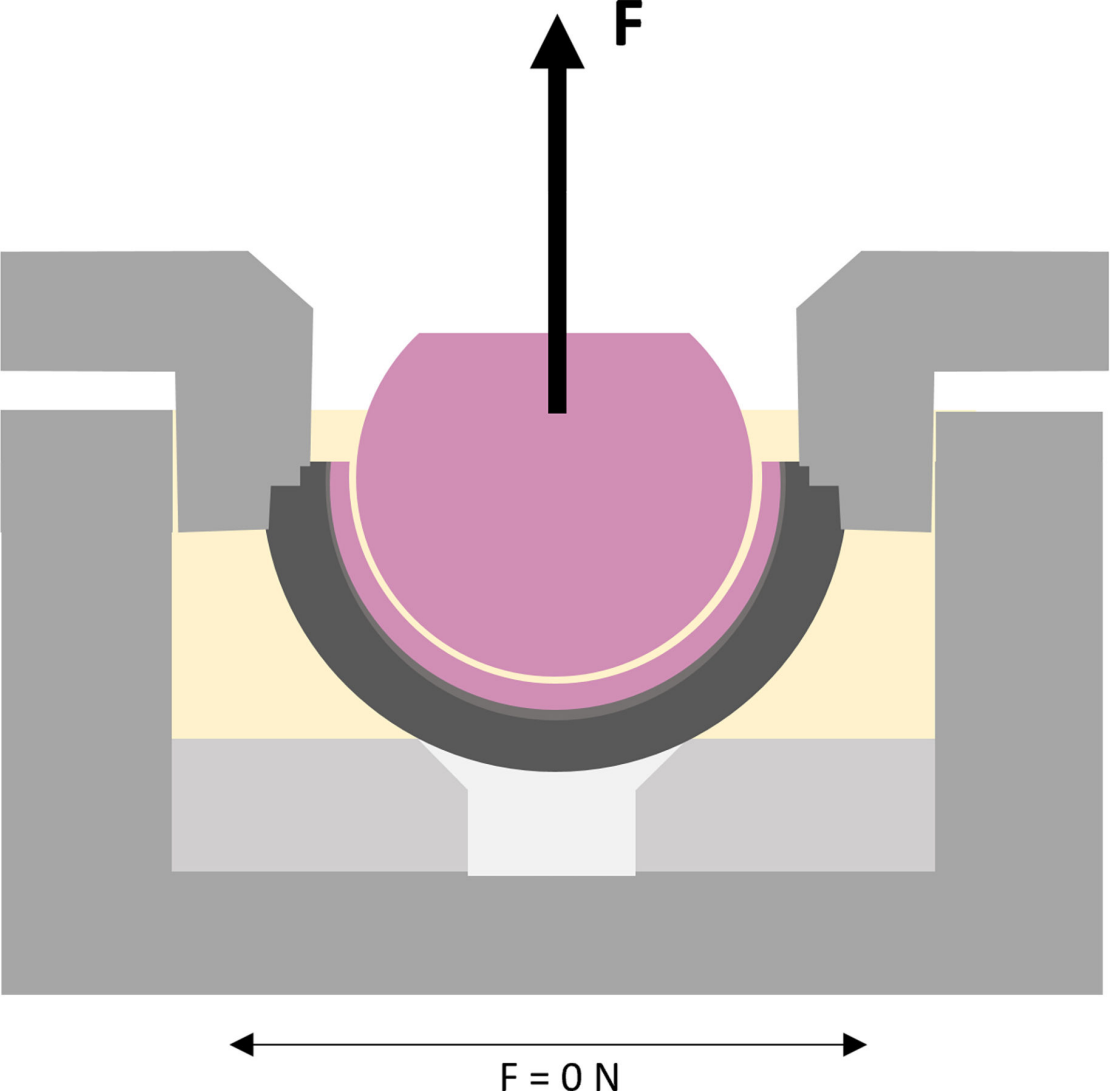
Test setup with fixed cup in serum on a transverse force balance. The femoral head is pulled out of the cup with a defined distraction speed.

The adhesion force of the head to the liner was analyzed depending on the distraction speed and the head size. A dedicated experimental setup was designed (Figure 2). The acetabular component, with the liner securely impacted with 2 kN according to the ASTM F1820-13,^28^ was held in place using a mounted fixture. Newborn calf serum with a protein concentration of 30 g/l was employed as the test medium, ensuring full coverage of the cup and liner. The femoral head was inserted into the liner with a standardized force of 20 N and subsequently extracted using a wire cable connected to a servo-hydraulic testing machine (MTS 858 Mini Bionix II; MTS Systems Corporation, USA) (Figure 2). This process was repeated five times for each configuration to ensure consistency in measurements. The distraction speed was varied incrementally from 10 mm/s to 120 mm/s in steps of 10 mm/s. The speed range was limited to 120 mm/s, as adhesion values plateaued at higher velocities, suggesting diminishing influence of further speed increases. A transverse force balance was used to counteract forces that do not act in the direction of tension.

**Fig. 3:**
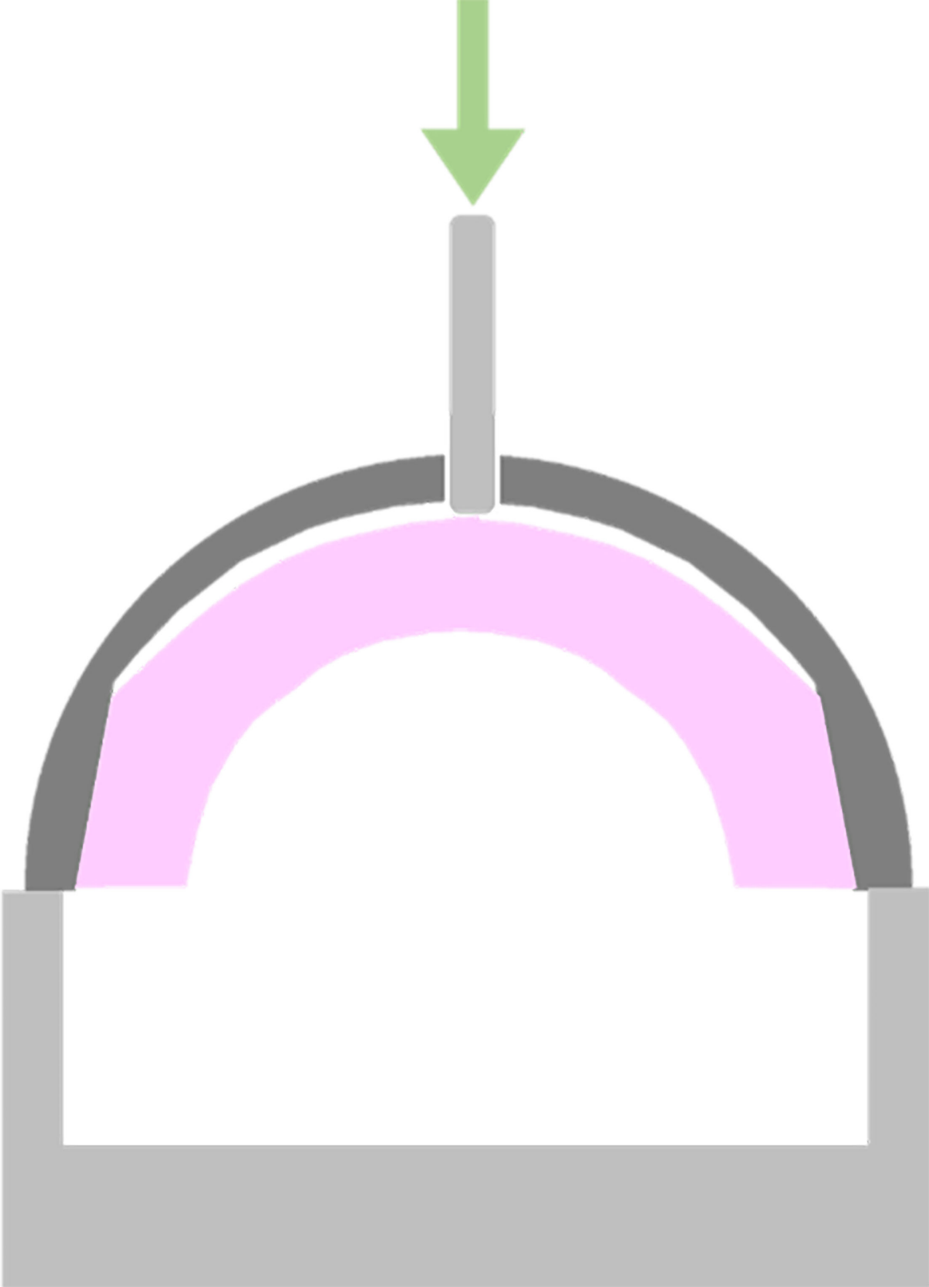
Setup for liner fixation force test.

In addition to the adhesion forces that are acting between the head and the liner, the liner fixation force, which is the force required to push the liner out of the cup, was determined. According to the standardized ASTM F1820-13,^28^ the liner was pressed orthogonally into the cup using a Zwick Z005 testing machine (Zwick Roell Group; Germany) at a controlled speed of 0.04 mm/s. To investigate the influence of the assembly force, forces of 1 kN, 2 kN, and 4 kN were applied. After assembly, axial disassembly was performed in accordance with the same standard. The liner was pushed out from the cup with a round rod at a speed of 5.1 cm/min according to ASTM F1820-13, and the maximum force required for disassembly was recorded (Figure 3).

### Statistical analysis

Descriptive data are presented as the arithmetic mean, SD, and range (minimum to maximum). The Shapiro-Wilk test was used to assess the normality of the data distribution. For the adhesion test, the Mann-Whitney U test was applied, while the liner fixation test was analyzed using one-way analysis of variance (ANOVA) with Bonferroni correction. A Pearson correlation coefficient was calculated to quantify the linear relationship between R' and force, using a one-tailed p-value to assess statistical significance. Statistical significance was defined as p < 0.05 for all tests. All analyses were performed using SPSS software (v. 25.0; IBM, USA).

## Results

### Equivalent radius

R' indicates the ratio of the head radius to the inlay radius. The Mathys system exhibited lower R' values than the DePuy system for both head sizes, with 4,172.3 mm and 8,268.0 mm for the 28 mm and 5,683.0 mm and 7,817.0 mm for the 36 mm for the Mathys and the DePuy System, respectively.

### Adhesion test for 28 mm ceramic heads

**Fig. 4:**
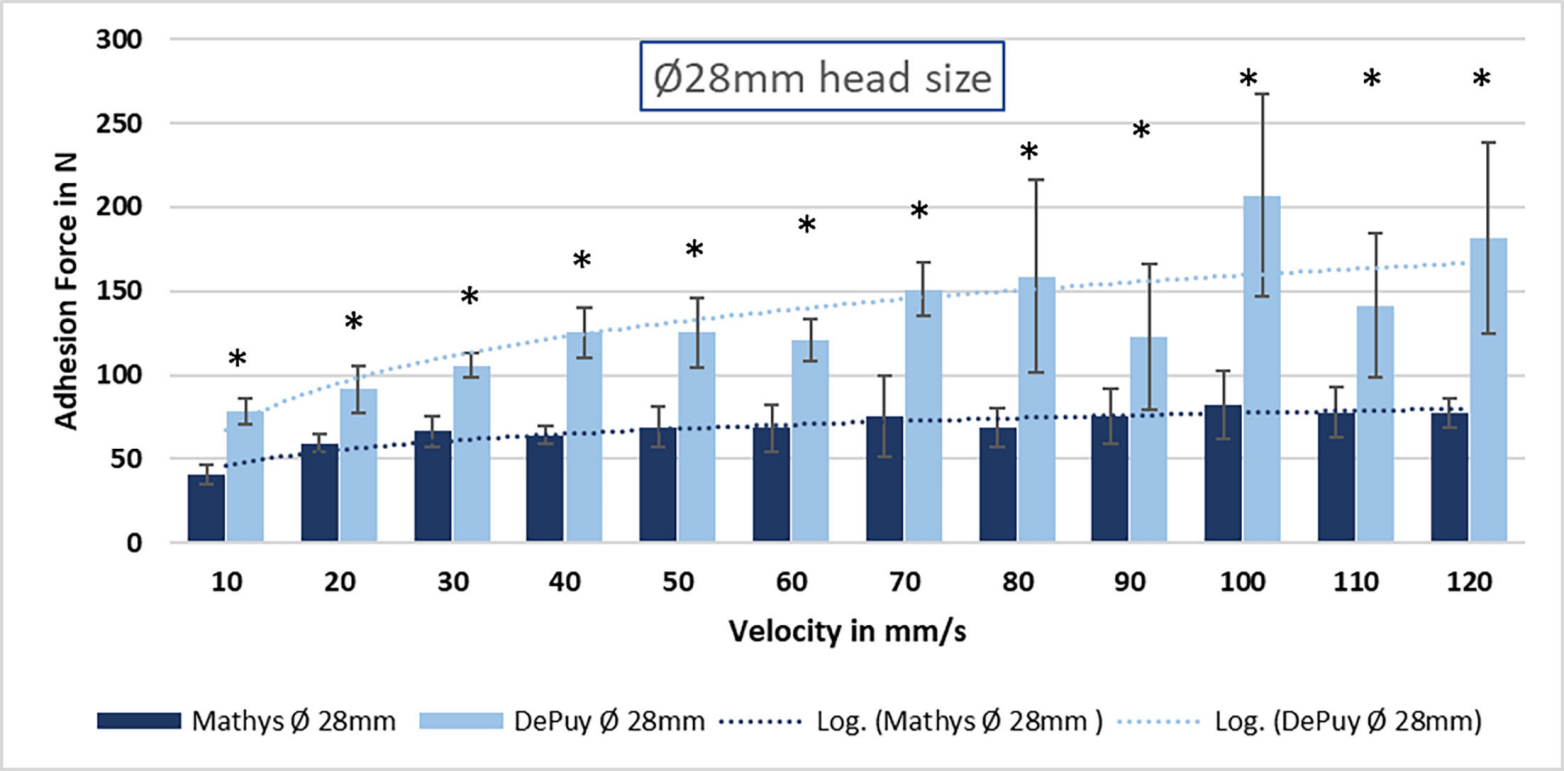
Adhesion force for 28 mm ceramic heads. *p < 0.05, Mann-Whitney U test.

The results for the 28 mm ceramic bearing demonstrate a logarithmic relationship between the velocity (10 to 120 mm/s) and the adhesion force for both implant systems (Mathys R² = 0.8811; DePuy R² = 0.6817). The DePuy bearing consistently exhibited higher adhesion forces compared to the Mathys bearing across all velocities, attributed to its greater R’ that promotes stronger adhesion between the ceramic head and liner (Figure 4, Table II). The DePuy system achieved a maximum adhesion force of 265.7 N, whereas the Mathys system reached 155.8 N.

**Table II.** Adhesion force for 28 mm ceramic heads.

Velocity, mm/s	Implant	Mean value F, N	SD	Min value F, N	Max value F, N	Mann-Whitney U statistic, Z statistic, and p-value
10	Mathys	40.6	5.7	30.5	50.7	U = 0.0, Z = -3.397, p < 0.001
	DePuy	78.6	7.6	68.9	90.3	
20	Mathys	59.5	5.7	50.5	72.9	U = 1.0, Z = -3.330, p < 0.001
	DePuy	91.4	13.7	69.9	105.4	
30	Mathys	66.4	9.0	54.1	96.9	U = 0.0, Z = -3.397, p < 0.001
	DePuy	105.6	7.2	98.6	114.7	
40	Mathys	64.1	5.3	56.4	73.2	U = 0.0, Z = -3.397, p < 0.001
	DePuy	125.3	15.0	105.3	140.5	
50	Mathys	69.2	12.2	55.4	96.9	U = 0.0, Z = -3.397, p < 0.001
	DePuy	125.1	21.1	106.9	154.2	
60	Mathys	68.4	14.1	54.3	114.4	U = 2.0, Z = -3.261, p < 0.001
	DePuy	120.6	12.3	105.6	135.2	
70	Mathys	75.4	23.9	57.6	155.8	U = 3.0, Z = -3.193, p < 0.001
	DePuy	151.1	15.6	129.3	171.4	
80	Mathys	68.4	11.5	54.2	106.2	U = 1.0, Z = -3.329, p < 0.001
	DePuy	158.8	57.1	97.3	221.2	
90	Mathys	75.2	16.5	58.8	111.9	U = 12.0, Z = -2.582, p = 0.007
	DePuy	122.5	43.4	71.5	168.6	
100	Mathys	82.4	20.4	62.5	140.3	U = 2.0, Z = -3.261, p < 0.001
	DePuy	206.7	60.2	114.3	265.7	
110	Mathys	77.8	14.7	60.6	115.4	U = 6.0, Z = -2.989, p = 0.001
	DePuy	141.3	42.9	84.6	187.8	
120	Mathys	77.3	8.5	66.7	103.7	U = 0.0, Z = -3.397, p < 0.001
	DePuy	181.3	56.9	129.1	246.7	

F, force.

### Adhesion force for 36 mm ceramic heads

**Fig. 5:**
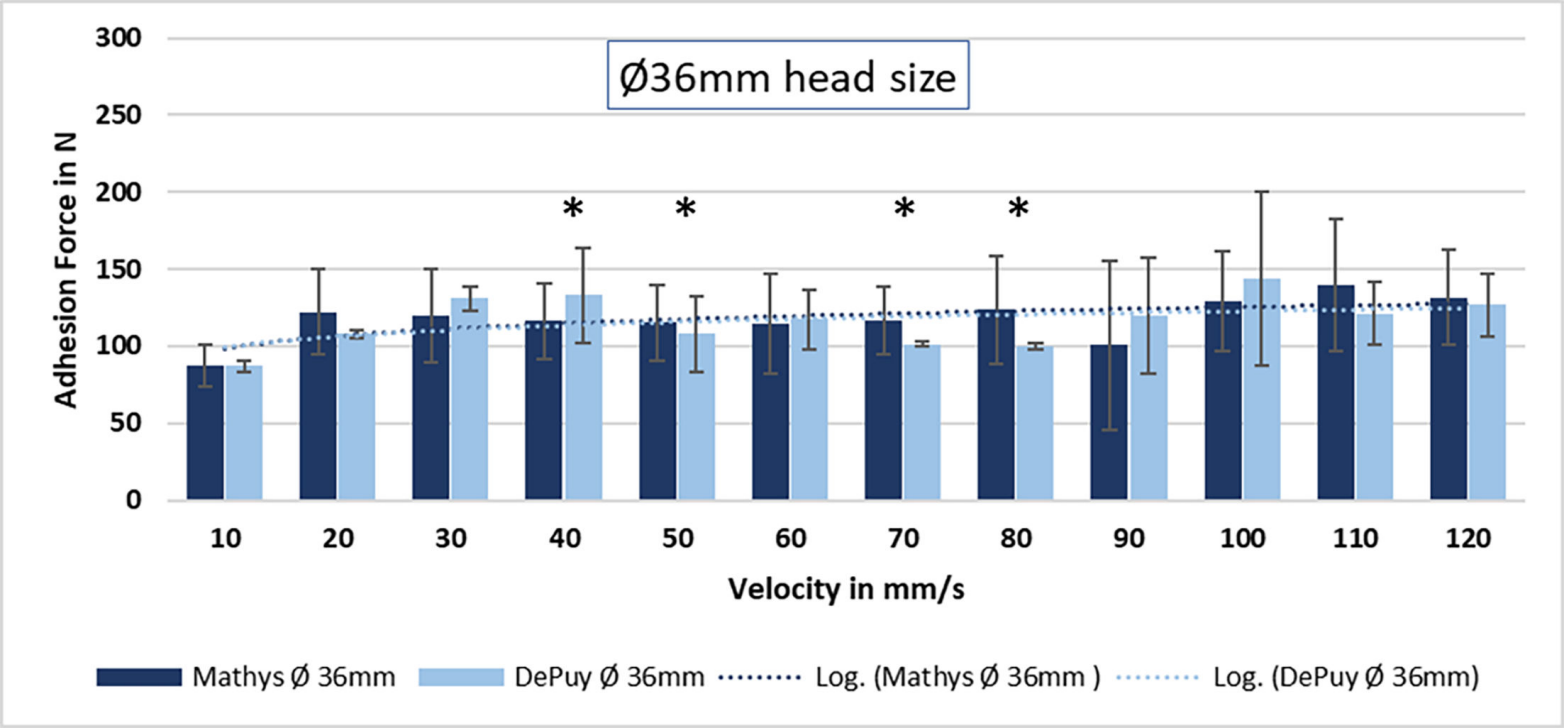
Adhesion force for 36 mm ceramic heads. *p < 0.05, Mann-Whitney U test.

Unlike the 28 mm heads, the adhesion forces for the 36 mm bearing remained relatively stable across all tested velocities, showing no significant logarithmic trend. Both implant systems exhibited comparable values, with maximum adhesion forces approaching 250.1 N for the Mathys System and 232.8 N for the DePuy System (Figure 5, Table III).

**Table III.** Adhesion force for 36 mm ceramic heads.

Velocity, mm/s	Implant	Mean value F, N	SD	Min value F, N	Max value F, N	Mann-Whitney U statistic,Z statistic, and p-value
10	Mathys	84.3	11.5	68.3	110.7	U = 179.0, Z = -0.509, p = 0.624
	DePuy	83.5	5.3	84.9	93.4	
20	Mathys	110.3	25.4	87.0	171.7	U = 195.0, Z = -0.121, p = 0.914
	DePuy	102.7	8.2	105.8	113.5	
30	Mathys	109.8	27.1	93.7	187.9	U = 120.0, Z = -1.940, p = 0.053
	DePuy	115.9	16.7	118.9	138.5	
40	Mathys	107.6	20.9	90.0	156.5	U = 104.0, Z = -2.328, p = 0.019
	DePuy	119.2	25.4	98.8	169.0	
50	Mathys	107.6	19.8	92.6	186.1	U = 108.0, Z = -2.231, p = 0.025
	DePuy	100.8	18.2	91.5	151.8	
60	Mathys	108.1	24.2	21.8	181.1	U = 191.0, Z = -0.218, p = 0.839
	DePuy	107.5	16.6	100.8	150.3	
70	Mathys	113.7	17.6	95.0	181.6	U = 101.0, Z = -2.401, p = 0.015
	DePuy	105.0	11.7	99.1	138.2	
80	Mathys	119.5	27.4	98.6	250.1	U = 31.0, Z = -4.099, p < 0.001
	DePuy	97.8	3.1	97.2	102.1	
90	Mathys	103.2	55.1	126.4	146.7	U = 131.0, Z = -1.673, p = 0.097
	DePuy	112.9	26.0	98.2	186.2	
100	Mathys	125.1	27.5	96.0	213.8	U = 144.0, Z = -1.358, p = 0.181
	DePuy	124.7	42.6	94.0	232.8	
110	Mathys	130.7	34.8	95.8	226.1	U = 175.5, Z = -0.594, p = 0.561
	DePuy	117.5	15.4	108.7	157.2	
120	Mathys	133.2	26.4	95.2	213.8	U = 137.0, Z = -1.528, p = 0.131
	DePuy	120.7	15.8	99.3	151.4	

F, force.

Based on the Pearson correlation coefficient, a statistically significant positive correlation between R’ over all head diameter and adhesion force is indicated by a one-sided p-value below the 0.05 threshold. However, no correlation was observed for the adhesion force at 100 mm/s. If the different head diameters are considered separately, a statistically significant positive correlation between R’ and the adhesion force was found at all tested velocities for the 28 mm head size. However, no significant correlation was found for the 36 mm head size at any tested velocity (Table IV).

**Table IV.** Pearson correlation analysis.

Velocity, mm/s	Pearson correlation overall	One-tailed significance	Pearson correlation (ø 28 mm)	One-tailed significance	Pearson correlation (ø 36 mm)	One-tailed significance
10	0.908	< 0.001	0.990	0.001	0.522	0.061
20	0.839	< 0.001	0.980	0.002	0.428	0.109
30	0.734	< 0.001	0.972	0.003	0.116	0.375
40	0.711	0.001	0.990	0.001	0.167	0.323
50	0.669	0.003	0.986	0.001	0.207	0.283
60	0.742	< 0.001	0.980	0.002	0.257	0.237
70	0.600	0.009	0.976	0.002	0.253	0.241
80	0.641	0.005	0.976	0.002	0.377	0.142
90	0.506	0.027	0.990	0.001	-0.045	0.451
100	0.374	0.085	0.977	0.002	-0.108	0.383
110	0.713	0.001	0.992	0.000	0.237	0.255
120	0.600	0.009	0.981	0.002	-0.008	0.491

### Liner fixation force with varying assembly forces

**Fig. 6:**
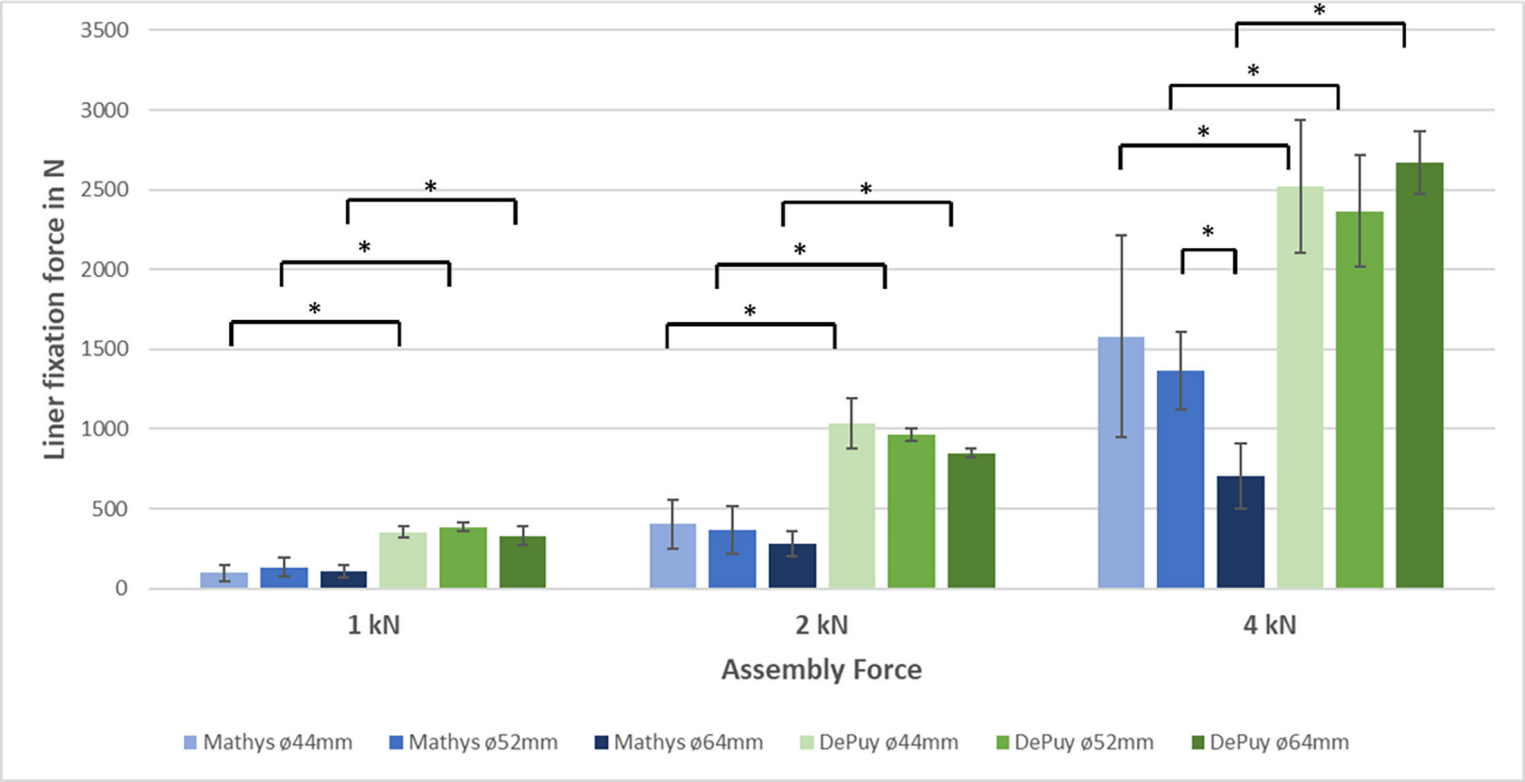
The liner fixation force of different cups/inserts.

The liner fixation force was examined as a dependency of the assembly force, the different liner sizes, and the liner design (Figure 6, Table V). A significant difference was found between the Mathys and DePuy liners for each assembly force as determined by one-way ANOVA (p < 0.001). DePuy showed significantly higher values for each liner size than Mathys.

**Table V.** Mean values, SDs, and minimum and maximum values of the liner fixation test.

Implant	Assembly force, kN	Mean value F, N	SD	Min value F, N	Max value F, N
Mathys Ø 44 mm	1	95.6	49.8	21.9	191.3
	2	402.9	152.2	119.9	570.1
	4	1578.3	633.7	493.0	2220.8
DePuy Ø 44 mm	1	354.1	33.1	316.4	388.6
	2	1032.6	156.5	851.4	1208.8
	4	2521.5	418.8	1953.8	2946.7
Mathys Ø 52 mm	1	131.4	59.1	30.5	189.4
	2	370.1	149.0	103.2	562.7
	4	1367.2	234.7	874.1	1737.2
DePuy Ø 52 mm	1	385.5	30.2	342.7	427.2
	2	962.7	40.7	923.7	1031.3
	4	2365.6	348.4	2024.2	2932.6
Mathys Ø 64 mm	1	108.5	41.7	40.6	179.2
	2	281.9	78.2	132.8	427.7
	4	705.6	203.3	371.1	1204.2
DePuy Ø 64 mm	1	329.7	60.1	225.2	375.2
	2	847.8	26.9	809.5	876.1
	4	2672.0	197.8	2464.6	2909.1

F, force.

As the assembly force increased, the liner fixation for both implant systems also increased. At 1 kN, assembly forces for all groups showed the lowest disassembly forces. While DePuy showed mean values of 354.1 N (Ø 44 mm), 385.5 N (Ø 52 mm), and 329.7 N (Ø 64 mm), Mathys had only achieved mean values of 95.6 N (Ø 44 mm), 131.4 N (Ø 52 mm), and 108.5 N (Ø 64 mm), which might be attributed to the different liner taper angles.

The highest liner fixation forces were measured at 4 kN assembly force. Average values of 1,578.3 N (Ø 44 mm), 1,367.2 N (Ø 52 mm), and 705.6 N (Ø 64 mm) were achieved for the Mathys system and 2,521.5 N (Ø 44 mm), 2,365.6 N (Ø 52 mm), and 2,672.0 (Ø 64 mm) for the DePuy system.

For the most part, a one-way analysis of variance revealed no significant difference between the various liner diameters, except at 4 kN between Mathys Ø 52 mm and Ø 64 mm (p < 0.001, one-way ANOVA). However, a trend can usually be seen that indicates that the liner fixation force decreases the larger the liner diameter becomes.

The Mathys system had minimum values of 21.9 N to 40.6 N at 1 kN assembly force. The DePuy system was above 225.5 N at each value. Maximum values were reached at 4 kN assembly force of 2,220.8 N (Mathys Ø 44 mm) and 2,946.7 N (DePuy Ø 44 mm).

## Discussion

Ceramic-on-ceramic bearings in hip arthroplasty achieve good clinical results, especially with regard to wear and the associated aseptic loosening.^1,2^ Nevertheless, clinical complications, such as squeaking noises, component failure, and even breakage, contribute to its limited clinical adoption.^8^ These issues are often linked to malalignment or incomplete fit of the ceramic liner.^9,10,13,14^ In addition to malpositioning during the surgical procedure, adhesion forces on the implant, which can lead to micro-separation,^18^ and eventually to micro-displacements between the liner and cup due to increased shear forces,^9^ may be a possible cause of postoperative malalignment of the liner. These adhesion forces can be provoked by subluxations of the prosthetic joint. Such a subluxation can occur during daily activities,^20-22^ physiotherapeutic treatments, or clinical functional tests of the prosthetic joint.^23,24^ In addition to the adhesion forces that occur, neck impingement can cause impaction to the liner, which can also lead to dislocation. In the present study, specific factors such as different implant designs, head diameter combined with the clearance between head and liner, dislocation speed, and the impaction force were investigated with regard to their influence on the potential dislocation of the ceramic liner.

The present investigations showed that relevant adhesion forces between the ceramic femoral head and the ceramic liner can occur. For heads with a diameter of 28 mm, the DePuy Synthes Pinnacle implant system achieved peak values of up to 265.7 N and the Mathys SeleXys system of 155.8 N. When using 36 mm head diameters, both systems achieved forces over about 200 N (DePuy Synthes Pinnacle: 232.8 N; Mathys SeleXys: 250.1 N).

Furthermore, the study reveals that there is a significant correlation between distraction speed and adhesion force in the case of smaller head sizes. The adhesion forces are higher with increasing speed. In addition, there is also a direct correlation with R’ between the smaller ceramic head and inlay. Here, a greater R’ leads to an increase in adhesion force. Neither correlation can be demonstrated with a larger head diameter. If the correlation is considered independently of the head diameter, a positive correlation between the radius difference and the head diameters can be seen across almost all speeds.

Comparable adhesion forces as measured in this study were also seen in experimental cadaver studies. Dienst et al^29^ investigated the traction force required on the leg to achieve dislocation of the hip joint. It was demonstrated that with a traction of 250 N, a mean distraction of the joint of 1.58 mm (SD 1.7) is already achieved. Wingstrand et al^30^ also showed in human specimens that a pulling force of 200 N can lead to a separation of the joint by up to 3 mm. In physiotherapeutic treatment, pulling forces are applied to mobilize the hip joint. Arvidsson^24^ investigated the influence of these pulling forces and showed that more than 400 N must be applied to achieve dislocation of the hip joint. Nevertheless, a separation of up to 1.4 mm is already measured with a tensile force of 200 N. Röling et al^31^ reported an average pulling force of 714 N required for dislocation. This leads to an average joint space widening of 8.8 mm. However, it is noted that the forces required depend on the positioning of the leg. These studies indicate that early postoperative physiotherapeutic therapy, for example, can lead to relevant pulling forces and that the adhesion forces between the head and liner can result in dislocation and subsequent malalignment of the liner. The tensile forces shown in the in vivo studies are not directly comparable with the in vitro results, as the influence of the surrounding tissue and muscles, as well as the interaction of adjacent joints, were not taken into account. Nevertheless, the separation of the joint caused by the tensile forces shows that this is clinically relevant. In addition, tensile forces may also be exerted on the leg during the operation, which can cause the liner to dislocate during the operation.

The liner fixation forces at different assembly forces showed that the measured adhesion forces are sufficient to dislocate the liner. At an assembly force of 1 kN, minimum values of 21.9 N were observed for the smallest cup diameter and 40.6 N for the largest diameter in the Mathys implant system. Both systems showed a correlation between the assembly force and liner fixation force. The higher the assembly force, the higher the fixation strength, emphasizing the importance of a sufficient assembly force to ensure implant stability. Regardless of the assembly force, the DePuy system consistently achieved higher values than the Mathys system. This may be due to the smaller cup angle of the DePuy system. The smaller cup angle contributes to better liner retention and resistance to disassembly. In a direct comparison of different cup diameters, there is no significant difference in fixation strength. Nevertheless, there is a slight trend toward a decrease in fixation force as the cup size increases. The results emphasize the importance of these design parameters in improving the mechanical performance of ceramic implant systems under varying disassembly and loading conditions.

The present study shows that when traction forces occur after surgery, the ceramic insert may be pulled out. Furthermore, low assembly forces are a relevant factor in this regard. Looking at the worst-case scenario of this study, the adhesion forces between the liner and head that occur may be sufficient to dislocate the inlay of the Mathys system when they are assembled with 1 kN. Even with an assembly force of 2 kN, the fixation forces would not be able to withstand the adhesive forces in some cases. With the DePuy system, dislocation may only occur with an assembly force of 1 kN and a large cup diameter. For the studied implants, an assembly force higher than 2 kN increases the probability that the insert is well fixed in the cup and will not come loose due to adhesion forces between the liner and head. Nevertheless, it should be noted that excessive assembly force may also have negative effects such as ceramic fractures or loosening of the cup.^32^ In order to achieve sufficient but not excessive impaction force clinically, a supporting surgical instrument could be used, such as the one recently developed and introduced for hip head impaction.^33-35^

For the experimental study, bovine serum was used at room temperature. The different viscosity and composition of human synovia may influence the adhesion forces between the components. Only two different implant designs were used to analyze the influence of the design on adhesion and liner fixation forces. To specify the influence of other implant designs more precisely, additional implants should be tested in further investigations. Although heads with diameter of 28 mm are used less frequently in clinical practice, they were considered the smallest option in this study in order to examine the greatest possible difference in head diameter. The influence of a head with diameter of 32 mm on adhesion forces should be examined in further studies. As already mentioned, the position of the components can affect the occurring adhesion forces. In this study, the optimal pulling direction for experimental investigation was selected. This does not correspond to the anatomical orientation of the components.

This study investigated the adhesion forces between the ceramic liner and the head, as well as the fixation strength of the ceramic liner in the cup, depending on the assembly force. The results showed that the occurring adhesion forces in combination with lower assembly forces can be high enough to induce a liner dislocation from the cup. Due to the adhesion forces in ceramic-ceramic bearings, there is a possibility that a dislocation of the liner from the cup may occur due to slight subluxation of the head. In order to prevent liner dislocation, an impaction of the liner with 2 kN or more is recommended for the investigated cups. However, other implant systems may have different thresholds.

## Data Availability

The data that support the findings for this study are available to other researchers from the corresponding author upon reasonable request.
